# Highly Fluorescent π-Conjugated Azomethines and Divalent Metal Complexes as Antibacterial and Antibiofilm Nominees

**DOI:** 10.1007/s10895-024-03855-x

**Published:** 2024-07-30

**Authors:** Şeyma Nur Ural Baydeniz, Halil İsmet Uçan, Fatih Sevgi, İhsan Obalı, Aslıhan Yılmaz Obalı

**Affiliations:** 1 Department of Chemistry, Science Faculty, Selcuk University, Konya, Türkiye; 2 Department of Medical Services and Techniques, Vocational School of Health Services, Selcuk University, Konya, Türkiye; 3 Department of Biology, Science Faculty, Selcuk University, Konya, Türkiye

**Keywords:** Hydroxy-substitution, Schiff base, Fluorescent, Antibiofilm, Antibacterial, MRSA, *Proteus mirabilis*

## Abstract

π-Conjugated azomethine ligands differing in the naphthalene or phenylmethane-centered core structure and their divalent cobalt, nickel, copper, and zinc metal complexes were prepared and well-characterized by spectral analyses in solid state. Magnetic natures of the complexes were determined by magnetic susceptibility measurements in solid-state. Their remarkable photophysical characteristics were recorded by Uv–vis and Fluorescence spectroscopic techniques. At their excitation wavelenght of 265 nm, all molecules exhibited triple fluorescence emission bands with promising intensities above 673 nm in near infra-red region. Antibacterial and antibiofilm activities of the π-conjugated azomethines are promising for potential applications in medical and healthcare settings. Hence, the antibacterial/antibiofilm activity of the π-conjugated azomethine ligands and their metal complexes against some clinically important bacteria namely *Staphylococcus aureus* (MSSA), Methicillin-resistant *Staphylococcus aureus* (MRSA), *Staphylococcus epidermidis*, *Escherichia coli*, *Pseudomonas aeruginosa* and *Proteus mirabilis* was investigated, and the obtained results have shown that the ligands and complexes had a remarkable antibacterial effect, especially on *Proteus mirabilis*. Metal complexes have been found to have a significant inhibitory effect on biofilm formation by MRSA, MSSA, and *P. mirabilis* compared to ligands. The copper (II) complex of ligand-2 showed the highest inhibition percentage, significantly reducing biofilm formation for MRSA and MSSA. Furthermore, cobalt (II) complexes of the ligands selectively inhibited the growth of the opportunistic pathogen *P. mirabilis* biofilms, indicating that metal complexes might be a good choice for future antibiofilm studies.

**Supplementary Information:**

The online version contains supplementary material available at 10.1007/s10895-024-03855-x.

## Introduction

‘Azomethines’ are an important part of ligands in coordination chemistry associated with their electron donor ability and Lewis base property by nature. They are nitrogen analogs of aldehyde or ketone with the replacement of the carbonyl group by the imine groups as CH = NR and stable products of condensation reaction between primary amines and aldehydes/ketones with mild conditions [1–3]. Azomethine compounds have occupied an important place in the photometric and fluorescent analysis. Especially, aromatic groups as π-electron delocating moiety in azomethine ligand design cause the formation of conjugated structure that contribute systems more fluorescent property [4–6]. π-Conjugated azomethines can improve the therapeutic profile of metal complexes by favorably modulating the hard/soft property of coordinating metal ions and the lipophilic/hydrophilic balance of the resulting complex. Hydroxy-substitutions to the conjugated azomethine ligands contribute multidentate area for coordination chemistry and biological studies. Hydroxy-substitued azomethine complexes construct a variety of new frame works with interesting bio-medical properties such as anti-inflammatory, antifungal, antioxidant, antibacterial, antibiofilm activities [7–10].

Among transition metals, divalent cobalt and copper complexes of azomethines show bioactive properties mostly. Cobalt ion is an essential for organisms is found in a variety of metalloproteins, vitamin B12 and required in the center of coenzymes and regulates nervous system. Copper is an element of fundamental importance for the formation and functioning of several enzymes and proteins and is required for cardiovascular integrity, lung elasticity, neovascularization, neuroendocrine function, and iron metabolism [11, 12]. Researches on these vitally important metal ions attract attention in bioactivity effect towards microorganisms in the context of metal ion-azomethine ligand bondings [13–16].

Nowadays in biological studies, biofilm formation and synthesis of antibiofilm agents have become a very important issue. Microbial biofilm is a rapidly reproducing community of bacteria, embedded in a self-producing matrix, forming on abiotic or biotic surfaces. Microbial infections are most often complicated by biofilm formation on prosthetic materials or natural tissues. Biofilm formation acts as an important self-defense strategy used by bacteria to protect itself against external changes and antibiotics effects. Moreover, biofilms have serious environmental and health impacts. There are numbers of bacteria responsible for biofilm related infections in the medical setting and responsible for nosocomial infections like *Proteus mirabilis* and *Staphylococcus aureus*. This increased biofilm formation and its resistance to conventional treatment enhances need to synthesize new drugs in the form of metal complexes. Antibacterial and antibiofilm activities of the Schiff bases and their metal complexes are promising in combating drug-resistant bacteria and preventing the formation of biofilms on medical devices [17–19].

For inhibition of biofilm of this rapidly reproducing microorganism, designing synthetic antibacterial drugs has also become an important research area [20–22]. Therefore, π-conjugated azomethines and their divalent copper and cobalt complexes could be used as suitable inhibitory agents for microorganism formations. These aspects are relevant with chemical design of azomethine compounds that they can engage a large variety of non-covalent interactions with biological targets, sucah as hydrogen bonding, dipole–dipole, lone pair effect rendering them powerful pharmacore [23, 24]. This study focused on π-conjugated azomethine ligands with electron rich naphthalene or phenylmethane center and their divalent cobalt, copper, nickel and zinc complexes are presented. Their promising fluorescence properties, antibacterial and antibiofilm activity studies are reported.

## Experimental

### Materials and Instrumentation for Chemical Studies

The reagents and chemicals were purchased from Sigma-Aldrich; 3,4-dihydroxybenzaldehyde, 4-aminobenzoic acid, 1,5-diaminonaphthalene, 4,4-diaminodiphenylmethane, 2-hydroxybenzaldehyde (salicylaldehyde), 3,4,5-trimethoxyaniline, Co(CO_2_CH_3_)_2_.2H_2_O, Ni(CO_2_CH_3_)_2_.4H_2_O, Cu(CO_2_CH_3_)_2_.2H_2_O, Zn(CO_2_CH_3_)_2_.2H_2_O, DMSO-d6 for NMR spectroscopy MagniSolv™.

^1^H-NMR and ^13^C-NMR spectra were recorded on a Varian 400-MHz Spectrometer and Bruker Biospin 300-MHz Spectrometer, respectively. FT-IR spectra were recorded on a PerkinElmer Spectrum 100 FT-IR spectrometer. Elemental analyses were carried out using a LECO-CHNS-932 elementel analyser. Melting points were determined by Büchi Melting Point B-540 instrument. Magnetic susceptibilities of metal complexes were determined using a Sheerwood Scientific MX Gouy magnetic susceptibility apparatus using the Gouy method with Hg[Co(SCN)]_4_ as calibrant. UV–vis spectra were recorded on PerkinElmer Lambda 25 UV–Vis Spectrometer. Fluorescence data were obtained by using a PerkinElmer LS 55 Luminescence Spectrometer. All measurements were carried out at 298 K.

### Synthesis Procedures

#### General Ligand Synthesis Procedures

General reaction procedure of the azomethine ligands have been based on Schiff base condensation as; different substitued aldehyde dervatives were reacted with different secondary amines.

##### (E)-4-((3,4-dihydroxybenzylidene)amino)benzoic acid 1

To a stirred solution of 3,4-dihydroxybenzaldehyde (1.38 g, 5 × 10^–2^ mol) in 5 mL methanol, the solution of 4-aminobenzoic acid (1.37 g, 5 × 10^–2^ mol) in 10 mL methanol was added to this mixture and continued to constant stirring for 6 h at 100 °C. The resulting crude product were filtered and recrystallized from methanol. Dried under vacuum to afford red solid.

M.P: 280 °C. FT-IR $\upsilon$(cm^−1^): 3304 (Ar-OH), 2836 (-COOH), 1670 (-C = O), 1643 (-C = N). ^1^H NMR (DMSO‐d6) δ(ppm): 12.52 (s, 1H, -COOH), 9.5 (s, 2H, Ar-OH), 8.4 (s, 1H, -CH = N), 6.9–8.2 (m, 7H, Ar–CH). ^13^C NMR (DMSO‐d6) *δ* (ppm): 169.3, 160.0, 157.2, 149.6, 146.1, 131.3, 131.6, 128.7, 123.2, 122.2, 117.4, 116.3. Analytical Calculation (%) for (C_14_H_11_NO_4_): C: 65.37, H: 4.31, N: 5.45; Found (%): C: 64.99, H: 4.29, N: 5.41.

##### 4,4'-((1***E***,1'***E***)-(naphthalene-1,5-diylbis(azaneylylidene))bis(methaneylylidene))bis(benzene-1,2-diol), 2

To a stirred solution of 3,4-dihydroxybenzaldehyde (2.76 g, 5 × 10^–2^ mol) 10 mL methanol, the solution of 1,5-dimainonaphtalene (1.58 g,, 2.5 × 10^–2^ mol) in 10 mL methanol was added to this mixture and continued to constant stirring for 4 h at 70 °C. The resulting crude product were filtered and recrystallized from methanol. Dried under vacuum to afford brown solid.

M.P: 270 °C. FT-IR $\upsilon$(cm^−1^): 3438 (Ar-OH), 1586 (-C = N). ^1^H NMR (DMSO‐d6) δ(ppm): 9.5 (s, 4H, Ar-OH), 8.4 (s, 2H, -CH = N), 6.8–8.3 (m, 12H, Ar–CH). ^13^C NMR (DMSO‐d6) *δ* (ppm): 160.0, 151.9, 149.6, 146.1, 131.3, 129.1, 126.8, 128.6, 123.2, 115.2, 116.3, 117.4. Analytical Calculation (%) for (C_24_H_18_N_2_O_4_): C: 72.35, H: 4.55, N: 7.03; Found (%): C: 72.32, H: 4.49, N: 7.00.

##### 4,4'-((1E,1'E)-((methylenebis(4,1-phenylene))bis(azaneylylidene))bis(methaneylylidene))bis- (benzene-1,2-diol), 3

To a stirred solution of 3,4-dihydroxybenzaldehyde (1.98 g, 5 × 10^–2^ mol) 10 mL methanol, the solution of 4,4-diaminodiphenylmethane (1.98 g, 2.5 × 10^–2^ mol) in 5 mL methanol was added to this mixture and continued to constant stirring for 4 h at 50 °C. The resulting crude product were filtered and recrystallized from methanol. Dried under vacuum to afford yellow solid.

M.P: 254 °C. FT-IR $\upsilon$(cm^−1^): 3499 (Ar-OH), 1629 (-C = N). ^1^H NMR (DMSO‐d6) δ(ppm): 9.5 (s, 4H, Ar-OH), 8.4 (s, 2H, -CH = N), 6.5–7.5 (m, 14H, Ar–CH), 3.95 (s, 2H, -CH_2_). ^13^C NMR (DMSO‐d6) *δ* (ppm): 160.0, 149.6, 149.5, 146.1, 140.0, 130.8, 131.3, 123.2, 122.8, 117.4, 116.3, 41.3. Analytical Calculation (%) for (C_27_H_22_N_2_O_4_): C: 73.96, H: 5.06, N: 6.39; Found (%): C: 73.91, H: 5.02, N: 6.35.

##### (E)-2-(((3,4,5-trimethoxyphenyl)imino)methyl)phenol, 4

To a stirred solution of 2-hydroxy- benzaldehyde (1.046 mL, 5 × 10^–2^ mol) 5 mL methanol, the solution of 3,4,5-trimethoxyaniline (1.83 g, 5 × 10^–2^ mol) in 15 mL methanol was added to this mixture slowly and continued to constant stirring for 8 h at 50 °C. The resulting crude product were filtered and recrystallized from methanol. Dried under vacuum to afford yellow solid.

M.P: 115 °C. FT-IR $\upsilon$(cm^−1^): 3079 (Ar-OH), 2824 (-CH_3_), 1611 (-C = N), 1123 (-O-CH_3_). ^1^H NMR (DMSO‐d6) δ(ppm): 13.2 (s, 1H, Ar-OH), 9.0 (s, 2H, -CH = N), 6.8–7.6 (m, 6H, Ar–CH), 3.92 (s, 6H, -OCH_3_), 3.81 (s, 3H, -OCH_3_). ^13^C NMR (DMSO‐d6) *δ* (ppm): 161.1, 160.0, 152.1, 147.5, 137.7, 132.1, 132.4, 121.4, 120.5, 117.8, 101.6, 60.8, 56.1. Analytical Calculation (%) for (C_16_H_17_NO_4_): C: 66.89, H: 5.96, N: 4.88; Found (%): C: 66.83, H: 5.89, N: 4.83.

#### General Metal Complexation Procedures

General synthesis procedures of the metal complexes were prepared by the reaction between corresponding ligands (1 equiv.) with some metal salts with Co(CO_2_CH_3_)_2_.2H_2_O, Ni(CO_2_CH_3_)_2_.4H_2_O, Cu(CO_2_CH_3_)_2_.2H_2_O, Zn(CO_2_CH_3_)_2_.2H_2_O (1 equiv.) separately with the followed procedure:

Azomethine ligands (5 × 10^–4^), **1** (0.128 g) / **2** (0.198 g) / **3** (0.219) / **4** (0.143) was dissolved in 15 mL methanol and added to the methanol solution of the 5 × 10^–4^ mol of metal salts of; Co(CO_2_CH_3_)_2_.2H_2_O 0.062 g for **1-Co**, 0.124 g for **2-Co**, 0.124 g for **3-Co**, 0.062 g for **4-Co**, Ni(CO_2_CH_3_)_2_.4H_2_O 0.062 g for **1-Ni**, 0.124 g for **2-Ni**, 0.124 g for **3-Ni**, 0.062 g for **4-Ni**, Cu(CO_2_CH_3_)_2_.2H_2_O 0.0495 g for **1-Cu**, 0.099 g for **2-Cu**, 0.099 g for **3-Cu**, 0.0495 g for **4-Cu**, Zn(CO_2_CH_3_)_2_.2H_2_O 0.0545 g for **1-Zn**, 0.109 g for **2-Zn**, 0.109 g for **3-Zn**, 0.0545 g for **4-Zn**, respectively. The mixtures were refluxed 3 days to give high yields, seperately. The resulting precipitates were collected and washed with cold methanol twice and cristallized by methanol with slow evaporation to give colored complexes.

**1-Co;** FT-IR $\upsilon$(cm^−1^): 3345 (Ar-OH), 3019 (-COOH), 1636 (-C = O), 1577 (-C = N), 432 (M–N). Analytical Calculation (%) for (C_32_H_28_CoN_2_O_12_): C: 55.58, H: 4.08, N: 4.05; Found (%): C: 55.43, H: 4.12, N: 4.01. **1-Ni;** FT-IR $\upsilon$(cm^−1^): 3380 (Ar-OH), 3154 (-COOH), 1641 (-C = O), 1581 (-C = N), 436 (M–N). Analytical Calculation (%) for (C_32_H_28_NiN_2_O_12_): C: 55.60, H: 4.08, N: 4.05; Found (%): C: 55.54, H: 4.02, N: 4.01. **1-Cu;** FT-IR $\upsilon$(cm^−1^): 3327 (Ar-OH), 3052 (-COOH), 1643 (-C = O), 1579 (-C = N), 430 (M–N). Analytical Calculation (%) for (C_32_H_28_CuN_2_O_12_): C: 55.21, H: 4.05, N: 4.02; Found (%): C: 55.19, H: 4.02, N: 4.04. **1-Zn;** FT-IR $\upsilon$(cm^−1^): 3366 (Ar-OH), 3067 (-COOH), 1643 (-C = O), 1543 (-C = N), 434 (M–N). Analytical Calculation (%) for (C_32_H_28_ZnN_2_O_12_): C: 58.00, H: 3.82, N: 4.83; Found (%): C: 57.99, H: 3.80, N: 4.88. **2-Co;** FT-IR $\upsilon$(cm^−1^): 3284 (Ar-OH), 1591 (-C = N), 451 (M–O). Analytical Calculation (%) for (C_32_H_30_Co_2_N_2_O_12_): C: 51.08, H: 4.02, N: 3.72; Found (%): C: 51.02, H: 4.01, N: 3.69. **2-Ni;** FT-IR $\upsilon$(cm^−1^): 3254 (Ar-OH), 1502 (-C = N), 448 (M–O). Analytical Calculation (%) for (C_32_H_30_Ni_2_N_2_O_12_): C: 51.11, H: 4.02, N: 3.73; Found (%): C: 51.09, H: 4.00, N: 3.78. **2-Cu;** FT-IR $\upsilon$(cm^−1^): 3078 (Ar-OH), 1632 (-C = N), 435 (M–O). Analytical Calculation (%) for (C_32_H_30_Cu_2_N_2_O_12_): C: 50.46, H: 3.97, N: 3.68; Found (%): C: 50.29, H: 3.94, N: 3.66. **2-Zn;** FT-IR $\upsilon$(cm^−1^): 3373 (Ar-OH), 1611 (-C = N), 426 (M–O). Analytical Calculation (%) for (C_32_H_30_Zn_2_N_2_O_12_): C: 50.22, H: 3.95, N: 3.66; Found (%): C: 50.19, H: 3.94, N: 3.63. **3-Co;** FT-IR $\upsilon$(cm^−1^): 3501 (Ar-OH), 1630 (-C = N), 405 (M–N). Analytical Calculation (%) for (C_35_H_34_Co_2_N_2_O_12_): C: 53.04, H: 4.32, N: 3.53; Found (%): C: 53.06, H: 4.31, N: 3.49. **3-Ni;** FT-IR $\upsilon$(cm^−1^): 3500 (Ar-OH), 1631 (-C = N), 407 (M–N). Analytical Calculation (%) for (C_35_H_34_Ni_2_N_2_O_12_): C: 53.08, H: 4.33, N: 3.54; Found (%): C: 53.11, H: 4.29, N: 3.59. **3-Cu;** FT-IR $\upsilon$(cm^−1^): 3500 (Ar-OH), 1630 (-C = N), 405 (M–N). Analytical Calculation (%) for (C_35_H_34_Cu_2_N_2_O_12_): C: 52.43, H: 4.27, N: 3.49; Found (%): C: 52.22, H: 4.19, N: 3.52. **3-Zn;** FT-IR $\upsilon$(cm^−1^): 3500 (Ar-OH), 1631 (-C = N), 406 (M–N). Analytical Calculation (%) for (C_35_H_34_Zn_2_N_2_O_12_): C: 52.19, H: 4.26, N: 3.48; Found (%): C: 52.21, H: 4.28, N: 3.52. **4-Co;** FT-IR $\upsilon$(cm^−1^): 3539 (Ar-OH), 1600 (-C = N), 465 (M–N), 440 (M–O). Analytical Calculation (%) for (C_32_H_34_CoN_2_O_8_): C: 60.67, H: 5.41, N: 4.42; Found (%): C: 60.66, H: 5.43, N: 4.40. **4-Ni;** FT-IR $\upsilon$(cm^−1^): Diss. (Ar-OH), 1618 (-C = N), 469 (M–N), 440 (M–O). Analytical Calculation (%) for (C_32_H_34_NiN_2_O_8_): C: 60.69, H: 5.41, N: 4.42; Found (%): C: 60.65, H: 5.44, N: 4.40. **4-Cu;** FT-IR $\upsilon$(cm^−1^): Diss. (Ar-OH), 1603 (-C = N), 466 (M–N), 441 (M–O). Analytical Calculation (%) for (C_32_H_34_CuN_2_O_8_): C: 60.23, H: 5.37, N: 4.39; Found (%): C: 60.19, H: 5.33, N: 4.41. **4-Zn;** FT-IR $\upsilon$(cm^−1^): Diss. (Ar-OH), 1600 (-C = N), 464 (M–N), 440 (M–O). Analytical Calculation (%) for (C_32_H_34_ZnN_2_O_8_): C: 60.05, H: 5.35, N: 4.38; Found (%): C: 60.02, H: 5.31, N: 4.39.

### Biological Activity

#### Bacterial Strains and Growth Media

The bacterial strains used were three Gram positive strains; *Staphylococcus aureus* (MSSA) ATCC 29213, Methicillin-resistant *Staphylococcus aureus* (MRSA) ATCC 43300, *Staphylococcus epidermidis* ATCC 35984 and three Gram negative strains; *Escherichia coli* ATCC 35218, *Pseudomonas aeruginosa* ATCC 27853, *Proteus mirabilis* ATCC 25933, obtained from the microorganism culture collection of Vocational School of Health Services (Selcuk University). Overnight cultures were grown routinely in Tryptic Soy Broth (TSB, Sigma-Aldrich) medium with shaking at 37°C. Mueller–Hinton Agar (MHA, Merck) and TSB was used for the Disc Diffusion and broth microdilution assay respectively. 96-well polystyrene flat bottom microtitration plates were purchased from Isolab.

#### Determination of Antibacterial Activity by Disc Diffusion Method

The modified Kirby-Bauer disc diffusion method was used to determine the antibacterial effects of the synthesized compounds [25]. Stock solutions of the synthesized compounds were prepared with DMSO at a concentration of 10 mg mL^−1^. After the MHA was prepared and sterilized, it was poured into sterile petri dishes in a thickness of 4 mm. Then turbidity of bacterial suspension was adjusted to 0.5 McFarland (≈10^8^cfu mL^−1^), and swabbed homogeneously on the plates using sterile cotton swabs. 15 µL solution of each compound was added to antibiotic-free paper discs. The petries were incubated at 37^0^C overnight and the zone diameters were determined carefully. The values are reported as the average of three measurements at Table 1.

**Table 1:** Antibacterial activities of compounds according to disc diffusion method†

	MSSA	MRSA	*S.epidermidis*	*E.coli*	*P.aeruginosa*	*P.mirabilis*
**1**	8	-	-	-	-	21
Ni	7	-	-	7	-	13
Co	13	10	11	9	9	10
Cu	7	-	-	-	-	12
Zn	8	8	8	-	-	15
**2**	11	10	12	-	7	20
Ni	7	-	-	-	-	9
Co	8	9	9	8	7	10
Cu	12	11	13	-	-	11
Zn	7	-	-	-	-	12
**3**	9	-	-	-	7	20
Ni	7	-	-	-	-	13
Co	7	7	8	-	7	-
Cu	8	-	-	-	-	18
Zn	8	7	-	-	-	15
**4**	-	-	-	-	-	-
Ni	-	-	-	-	-	-
Co	-	-	-	-	-	-
Cu	-	-	-	-	-	-
Zn	-	-	-	-	-	-

†: The values indicate the diameters (mm) of the inhibition zones.

-: No inhibition

#### Determination of Minimum Inhibitory Concentration (MIC)

The MIC was calculated by standard protocol of NCCLS document M7-A3 by 96-well plate microdilution method [26]. Briefly, cells were grown overnight at 37 °C to obtain single bacterial colonies and re-suspended in a 0.9% normal saline solution to give a turbidity equivalent to a 0.5 McFarland. The cells were then diluted to 100-fold in TSB media to give concentration of ≈10^6^ cfu/mL. The diluted cell suspensions were added to the wells of 96-well microtiter plates (100 μl/well) containing equal volumes of two-fold serial dilutions of compounds were prepared in TSB starting from a stock solution of compounds (4,096 mg/ml DMSO). In this manner final concentration of compounds range from 1024, 512, 256,…to 2 μg/mL and 5 × 10^5^ cfu/ mL bacteria in each well (last two wells are bacteria without any treatment and broth only control well respectively). The antibiotics were used as a positive control at 128 μg/mL in the first well. Then, the prepared plates were incubated at 37°C for overnight and the growths were measured at 600 nm by using a microplate reader (µQuant, BioTek). The MIC values were determined as the minumum concentration of compounds whose Optical Density (OD) values were comparable to the negative control wells [27]. The experiments were performed in triplicate on separate days for confirmation analysis and reproducibility. The MIC values are reported as the mean of three experiments at Table 2.

**Table 2:** Antibacterial activities of the compounds by Microdilution Broth Method (MIC, μg /mL)

	**MSSA**	**MRSA**	***S.epidermidis***	***P.mirabilis***
1	1024	1024	1024	256
Ni	1024	1024	1024	512
Co	256	512	512	256
Cu	1024	1024	1024	512
Zn	512	512	512	512
2	512	512	512	256
Ni	512	1024	512	256
Co	512	512	512	256
Cu	256	256	128	256
Zn	512	512	512	512
3	1024	1024	1024	256
Ni	1024	1024	1024	512
Co	1024	512	512	512
Cu	512	512	512	256
Zn	512	1024	512	512
CFR	2	8	16	< 0,25
CFZ	0,5	0,5	2	2

#### Antibiofilm Activity by Crystal Violet Assay

The microtiter plate based crystal violet assay described by Christensen et al. is widely used and is considered the standard test for the detection of biofilm formation [28]. Biofilm inhibition study was carried out by modifying Christensen's method on three staphylococcus strains and Proteus mirabilis known to produce biofilm [29, 30]. Briefly, the procedure we used above for MIC detection was followed using TSB with 1% glucose as a medium. The microplates are inverted and emptied after MIC measurement, and each well is washed three times with 250 µL sterile PBS using an automatic dispenser (BioTek Microfill). Thus, the planktonic bacteria are removed completely. The adherent sessile cells were stained with 225 μL of 0.1% crystal violet solution (prepared with sterile water and filtered with 0.45 µm sterile syringe tip) and incubated for 15 min at room temperature. After incubation, the staining solution was discarded and the wells were washed 3 times with 250 µL distilled water by help of automatic dispenser. After the plates were dried, the stained biofilm layer was dissolved with 250 µL 33% glacial acetic acid and OD (Optical Density) was measured with a microplate reader (BioTek µQuant) at a wavelength of 570 nm. The positive control is the combination with culture medium and bacteria, the negative control is culture media only. For each experiment, background staining was corrected by subtracting the crystal violet bound to negative controls (Blank) from those of the tested sample. Inhibitor-mediated reduction of biofilm formation was assessed by comparing it to the positive control. The biofilm assay was performed three times. The percentage reduction in biofilm was calculated by using following formula: Percent reduction in biofilm = [(Positive Control OD570 nm –Test OD570 nm)/Positive Control OD570 nm] × 100.

## Result and Discussion

### Synthesis and Characterizations

Target hydroxy-substitued azomethine ligands (E)-4-((3,4-dihydroxybenzylidene)amino)benzoic acid, **1**, 4,4'-((1*E*,1'*E*)-(naphthalene1,5diylbis(azaneylylidene))bis(methaneylylidene))bis(benzene-1,2-diol), **2**, 4,4'-((1E,1'E)-((methylenebis(4,1-phenylene))bis(azaneylylidene))bis(methaneylylidene))bis (benzene-1,2-diol), **3**, (E)-2-(((3,4,5-trimethoxyphenyl)imino)methyl)phenol,**4** and their divalent cobalt, nickel, copper, zinc metal complexes were reported here. Azomethine ligands were synthesized by Schiff base condensation reactions in methanol medium under reflux and obtained precipitates were collected and purified by simple filtration and drying methods.

**Scheme 1:**
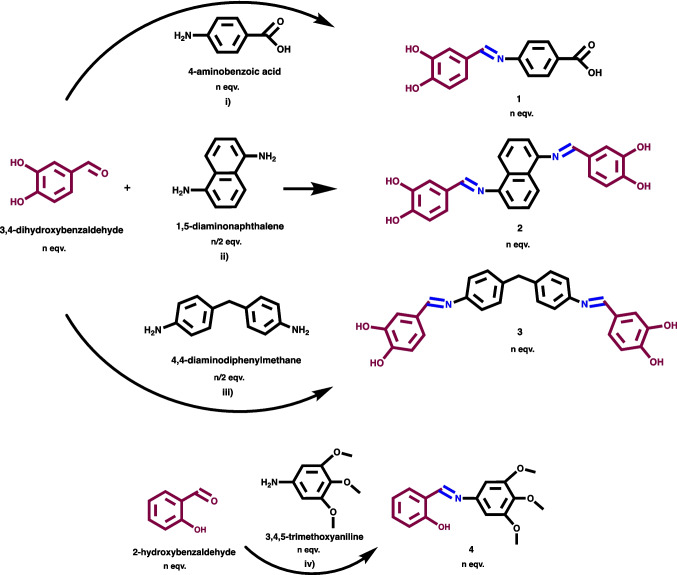
Synthesis routes of azomethine ligands; (E)-4-((3,4-dihydroxybenzylidene)amino)benzoic acid, **1**, 4,4'-((1*E*,1'*E*)-(naphthalene-1,5-diylbis(azaneylylidene))bis(methaneylylidene))bis(benzene-1,2-diol), **2**, 4,4'-((1E,1'E)-((methylenebis(4,1-phenylene))bis(azaneylylidene))bis(methaneylylidene))bis(benzene-1,2-diol), **3**, (E)-2-(((3,4,5-trimethoxyphenyl)imino)- methyl)phenol, **4** with the reaction contidions as; **i)** methanol, 6 h, 100 ^ο^C. **ii)** methanol, 4 h, 70 ^ο^C. **iii)** methanol, 4 h, 50 ^ο^C. **iv)** methanol, 8 h, 50 ^ο^C

Synthesis routes were presented in Scheme 1 as azomethines were generated from the condensation of primary amines and active carbonyl group of aldehyde species by nucleophilic addition with the result of a dehydration to generate an imine. **1**, **2** and **3** were obtained by the reaction of 3,4-dihydroxybenzaldehyde with the amine derivatives as 4-aminobenzoic acid/1,5-diaminonaphthalene/4,4-diaminodiphenylmethane, respectively. Last condensation reaction was performed by 2-hydroxybenzaldehyde and 3,4,5-trimethoxyaniline to give** 4**. Metal complexation reactions were performed in methanol medium under reflux.

Remarkable chemical and photophysical properties could be explained by their possible *e-donor binding sites* of designed azomethine ligands. They have *O,N-type* or *O,O-type* binding sites, those can successfully coordinate to metal ions and have promising photophysical properties. Their proposed structural data were elucidated by FT-IR spectroscopy, ^1^H-NMR spectroscopy, elemental analysis, melting point measurements.

**Fig. 1:**
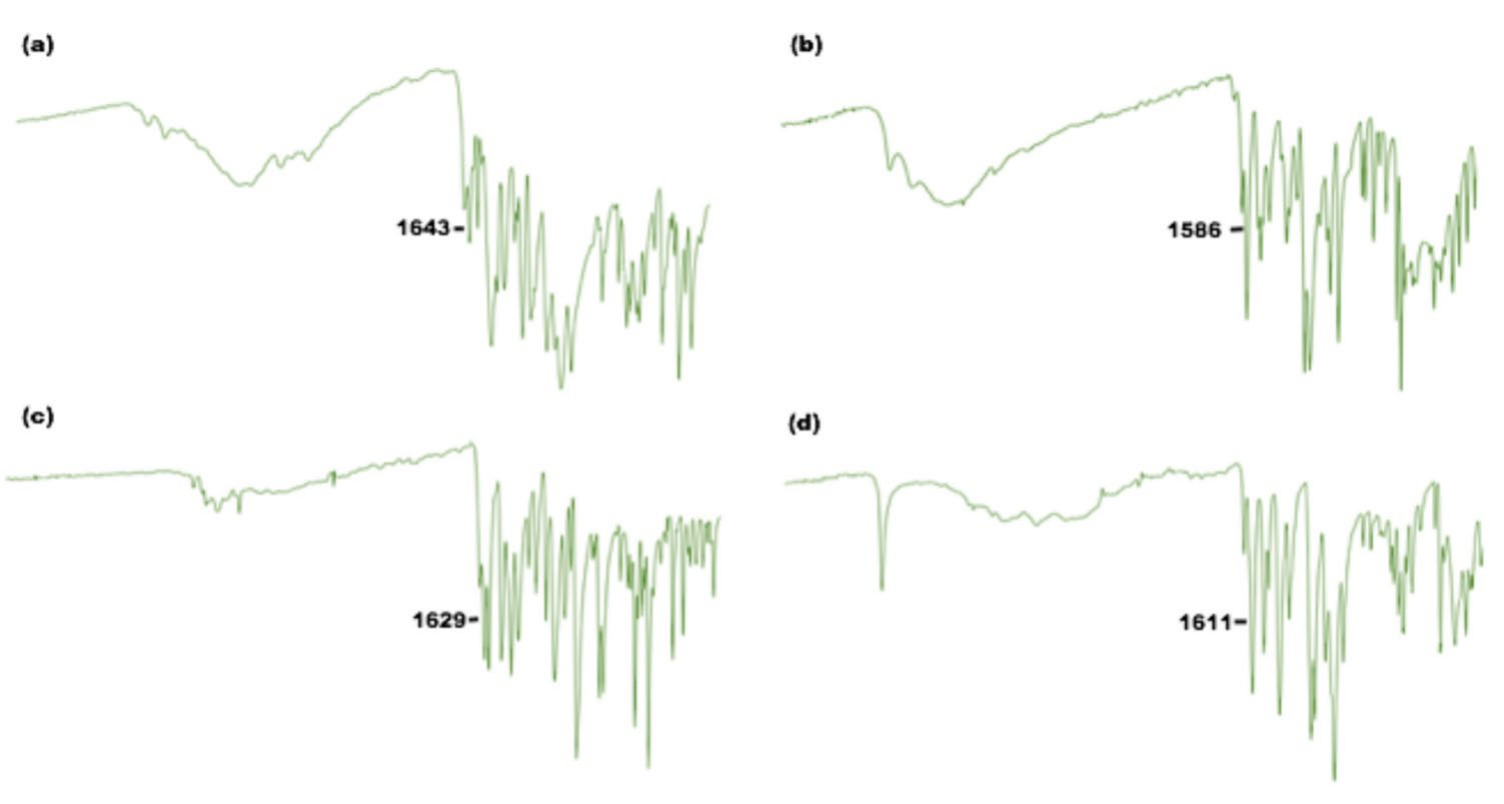
FT-IR spectra with C = N vibrations marked of azomethine ligands **a)** 1, **b)** 2, **c)** 3, **d)** 4

Prominent absorption bands belong to specific functional groups of azomethine ligands and metal complexes were observed in the FT-IR spectra (Fig. 1 and Fig. S1-S20). The formation of the π-conjugated azomethine ligands (**1**–**4**) were proved by the data of initial reagents. Disappear of the sharp amine double bands at 3460–3359 cm^−1^ which were belong to initial compound 4-aminobenzoic acid indicate the formation of azomethine ligand **1.** This shows nitrogen in the -NH_2_ group of 4-aminobenzoic acid turns into -C = N- imine nitrogen at 1643 cm^−1^. Additionally, the absorption band at 3304 cm^−1^ assigned to phenolic-OH stretch, 2836 cm^−1^ observed for carboxilic acid-OH stretch and 1670 cm^−1^ is for carboxilic acid-C = O for **1**. When the spectra of **2** was examined, it was not observed the the -NH_2_ band in 1,5-diaminonaphthalene, which constitutes the ligand. At the same time, the -C = O band of the aldehyde, which should be seen at 1700 cm^−1^, was observed at 1586 cm^−1^ because it turned into the C = N band. From the spectra of the **3**, the band of -C = O of the aldehyde, which was expected to be at 1700 cm^−1^, was observed at 1629 cm^−1^ as it turned into the C = N band. In the case of phenolic -OHs, it was observed at 3499 cm^−1^. As examined the spectra of the last azomethine ligand **4**, 1611 cm^−1^ band was observed due to the transformation of the C = O band of the aldehyde, which was expected to be in the range of 1700 cm^−1^, into the C = N band. At different positions, strong -O-CH_3_ etheric band was observed at 1123 cm^−1^ and -CH_3_ band was observed at 2824 cm^−1^.

After the complexation via the imine nitrogen or hydroxyl group in chelation with metal ions it was observed that the bands of the possible binding reigons were shifted or disapperad. Characteristic bands of the metal complexes were sorted out and some changes were reported here. From the spectra of **1** and the complexes, it was observed that -C = N- band of **1** at 1670 cm^−1^ was shifted to 1577 cm^−1^ for **1-Co**, 1581 cm^−1^ for **1-Ni**, 1579 cm^−1^ for **1-Cu**, 1585 cm^−1^ for **1-Zn**. Phenolic-OH band of **2** at 3438 cm^−1^ displayed shifts after complation to; 3284 cm^−1^ for **2-Co**, 3254 cm^−1^ for **2-Ni**, 3078 cm^−1^ for **2-Cu**, 3373 cm^−1^ for **2-Zn**. From the spectra of **3** and the complexes, it was observed that -C = N- band of **3** at 1629 cm^−1^ was shifted to 1630 cm^−1^ for **3-Co**, 1631 cm^−1^ for **3-Ni**, 1630 cm^−1^ for **3-Cu**, 1631 cm^−1^ for **3-Zn**. After complexations of **4** with metals, phenolic-OH band at 3079 cm^−1^ was shifted to higher frequencies at 3539 cm^−1^ for **4-Co** and was disappeared for **4-Ni**, **4-Cu** and **4-Zn**. And -C = N- bands at 1611 cm^−1^ was also shifted to; 1600 cm^−1^ for **4-Co**, 1618 cm^−1^ for **4-Ni**, 1603 cm^−1^ for **4-Cu,** 1600 cm^−1^ for **4-Zn**.

Complexations were also confirmed by the appearance of the new bands. Absorption bands at 432 cm^−1^, 436 cm^−1^, 430 cm^−1^, 434 cm^−1^ for **1-Co**, **1-Ni**, **1-Cu**, **1-Zn** represent metal-nitrogene vibrations; at 451 cm^−1^, 448 cm^−1^, 435 cm^−1^, 426 cm^−1^ for **2-Co**, **2-Ni**, **2-Cu**, **2-Zn** represent metal-oxygene vibrations; at 405 cm^−1^, 407 cm^−1^, 405 cm^−1^, 406 cm^−1^ for **3-Co**, **3-Ni**, **3-Cu**, **3-Zn** represent metal-nitrogene vibrations; 440 cm^−1^ and 465 cm^−1^; 469 cm^−1^ and 440 cm^−1^; 466 cm^−1^ and 441 cm^−1^; 464 cm^−1^ and 440 cm^−1^ which represent metal-nitrogene (M–N) and metal-oxygene (M–O) vibrations for **4-Co**, **4-Ni**, **4-Cu**, **4-Zn** complexes, respectively. Metal–Ligand bond frequencies are compatible with previous works [31, 32].

**Fig. 2:**
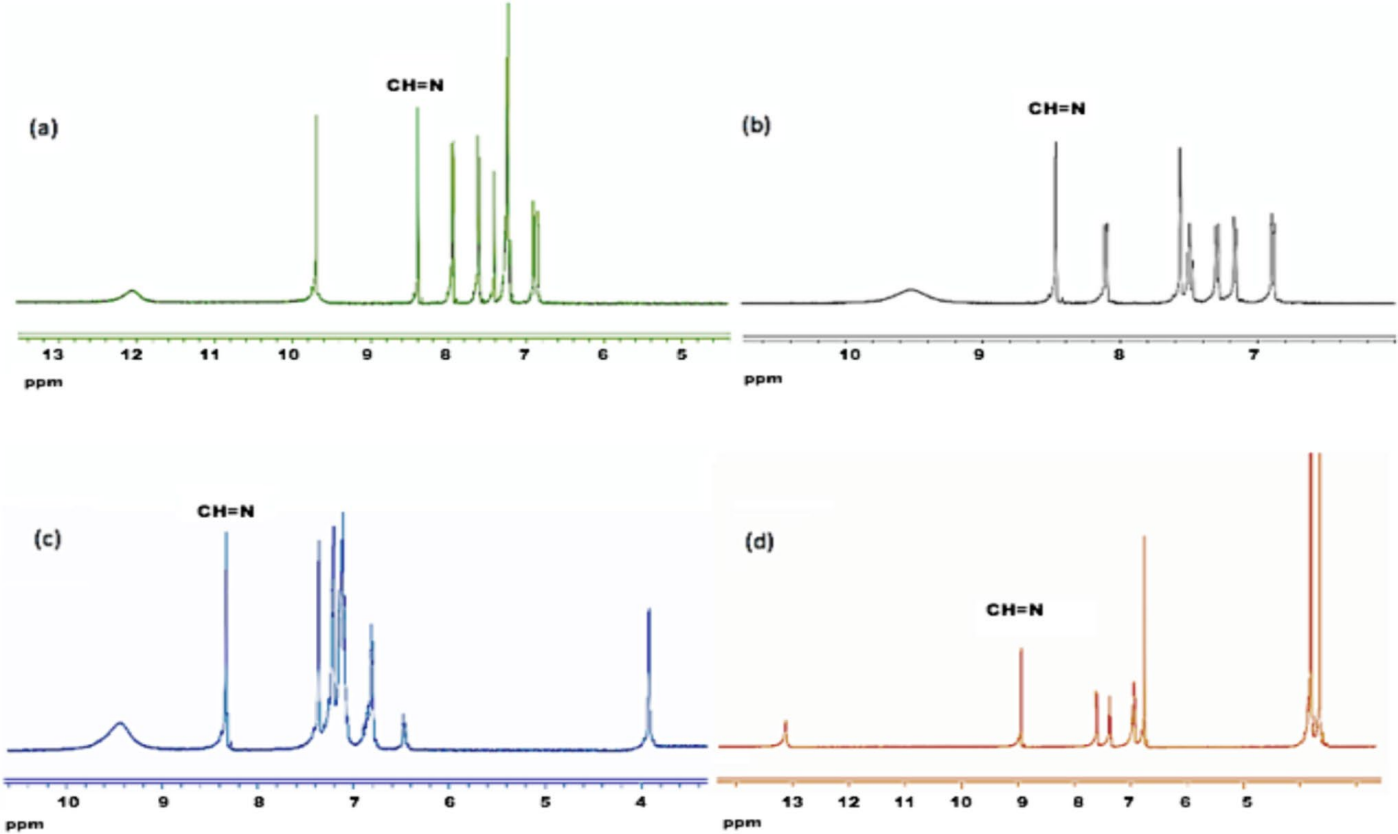
^1^H-NMR spectra with CH = N proton marked of azomethine ligands **a)** 1, **b)** 2, **c)** 3, **d)** 4

The ^1^H NMR spectra were in DMSO‐d6 and recorded (Fig. 2 and Fig. S21-S24). Formation of **1**, was confirmed by the singlet peak observed at 8.4 ppm corresponds to azomethine proton (-CH = N). This is also the same for other ligands as; azomethine singlet protons were observed at 8.4 ppm for **2**, 8.4 ppm for **3**, 9.0 ppm for **4**. The corresponding ^13^C NMR spectra were provided in Fig. S25-S28. Peaks at 160 ppm indicate azomethine carbons for all the azomethine ligands.

Magnetic susceptibility measurements were performed to express magnetic behaviours of all the metal complexes at ambient temperature. According to the results; nickel and zinc complexes were found to be diamagnetic and cobalt and copper complexes were found to be paramagnetic. The magnetic susceptibility measurement displayed negative signals which indicate diamagnetism for the complexes nickel and zinc complexes as expected. Theoretical calculations of magnetic moments (μ_eff_) of all the paramagnetic cobalt and copper complexes were found; 1.89 BM corresponding to one unpaired electron. These values are consistent with the expected spin-only magnetic moments (μ_eff_) as 1.73 BM [33, 34].

### Photophysical Properties

#### Absorption Spectra

**Fig.3:**
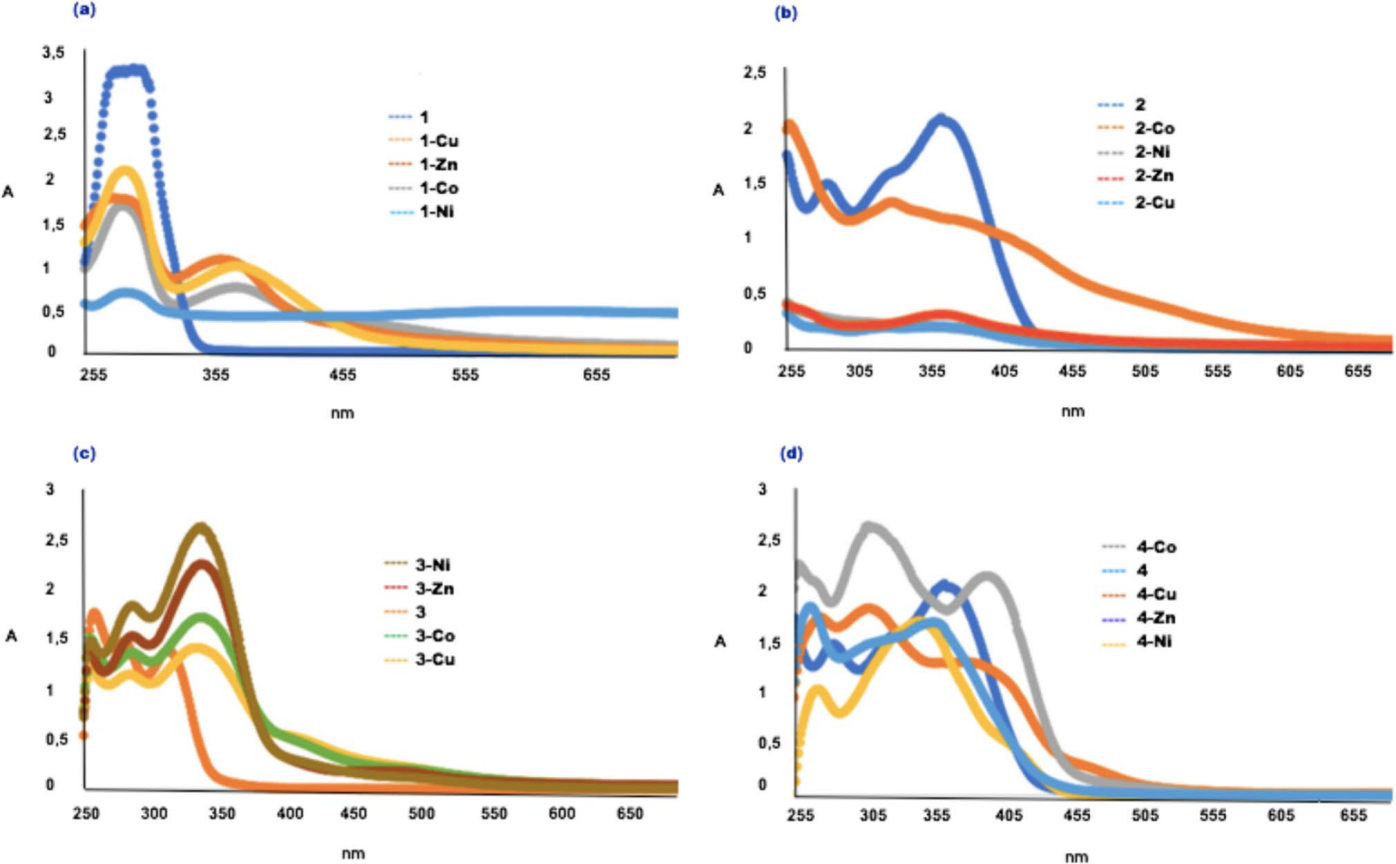
Absorption spectra of azomethines **1**, **2**, **3**, **4** and their Co(II), Ni(II), Cu(II), Zn(II) complexes

The Uv–Visible spectra of the azomethine ligands and their complexes (1 × 10^–4^ M) have been recorded in DMSO:Acetonitrile (ratio 1:1) at room temperature (Fig. 3). The intensive spectral bands were recorded at 293 nm (for **1**), 296 nm and 381 nm (for **1-Co**), 296 nm and 356 nm (for **1-Ni**), 294 nm and 382 nm (for **1-Cu**), 286 nm and 370 nm (for **1-Zn**); 287 nm and 370 nm (for **2**), 333 nm and 380 nm (for **2-Co**), 303 nm and 335 nm (for **2-Ni**), 290 nm and 366 nm (for **2-Cu**), 276 nm and 368 nm (for **2-Zn**); 260 nm, 280 nm and 311 nm (for **3**), 260 nm, 292 nm and 341 nm (for **3-Co**), 260 nm, 292 nm and 341 nm (for **3-Ni**), 260 nm, 292 nm and 341 nm (for **3-Cu**), 260 nm, 292 nm and 341 nm (for **3-Zn**); 262 nm, 282 nm and 368 nm (for **4**), 262 nm, 316 nm and 400 nm (for **4-Co**), 277 nm, 332 nm ve 350 nm (for **4-Ni**), 273 nm, 326 nm ve 400 nm (for **4-Cu**), 271 nm, 315 nm ve 361 nm (for **4-Zn**) belong to n → π* and π → π* transitions of intra-ligand charge transfer between conjugated aromatic groups and azomethine group in a system. From the spectra of metal complexes, the bands emerged above 350 nm which were assigned to π → π* transitions confirm the formation of coordination complex between azomethine ligand and the metal ion during the interaction. From the absorption spectra of the ligands and complexes it was observed as, **1, 2** have highest absorptions compared to their metal complexes. The absorption bands of the metal complexes of** 3** shifted towards higher wavelengths as hypsochromic shifts and increased in intensity compared to free ligands. **4-Co** has the highest absorption compared to trimethoxy-ended azomethine ligand **4** and its other complexes. Additionally, while the absorption bands of **4-Co**, **4-Cu**, **4-Zn** complexes showed hypsochromic shifts, **4-Ni** complexes only showed bathochromic shift in comparison of ligand **4**. During this coordination the lone pair of electrons present on azomethine nitrogen is transferred to the metal ion and so is not available for conjugation with the aromatic ring. ε values of the complexes were calculated from the formula of ε = Amax/l.C (l = 1 cm); where ε is the absorptivity coefficient also known as the extinction coefficient of the sample. It is a unique physical constant of the chemistry of the sample that relates to the sample's ability to absorb light at a given wavelength. A_max_ is maximum absorbance, C is molarity and ε values were calculated as; 32450 M^−1^ cm^−1^ for **1** at 293 nm, 16990 M^−1^ cm^−1^ for **1-Co** at 296 nm,6730 M^−1^ cm^−1^ for **1-Ni** at 296 nm, 20250 M^−1^ cm^−1^ for **1-Cu** at 294 nm, 17620 M^−1^ cm^−1^ for **1-Zn** at 286 nm, 20160 M^−1^ cm^−1^ for **2** at 370 nm, 12970 M^−1^ cm^−1^ for **2-Co** at 333 nm,2450 M^−1^ cm^−1^ for **2-Ni** at 303 nm, 2210 M^−1^ cm^−1^ for **2-Cu** at 290 nm, 3060 M^−1^ cm^−1^ for **2-Zn** at 368 nm, 16870 M^−1^ cm^−1^ for **3** at 260 nm, 16890 M^−1^ cm^−1^ for **3-Co** at 341 nm, 25630 M^−1^ cm^−1^ for **3-Ni** at 341 nm, 13760 M^−1^ cm^−1^ for **3-Cu** at 341 nm, 22070 M^−1^ cm^−1^ for **3-Zn** at 341 nm, 20380 M^−1^ cm^−1^ for **4** at 368 nm, 25,850 M^−1^ cm^−1^ for **4-Co** at 316 nm, 16650 M^−1^ cm^−1^ for **4-Ni** at 350 nm, 17320 M^−1^ cm^−1^ for **4-Cu** at 326 nm, 17640 M^−1^ cm^−1^ for **4-Zn** at 271 nm.

#### Fluorescent Emission Studies

**Fig.4:**
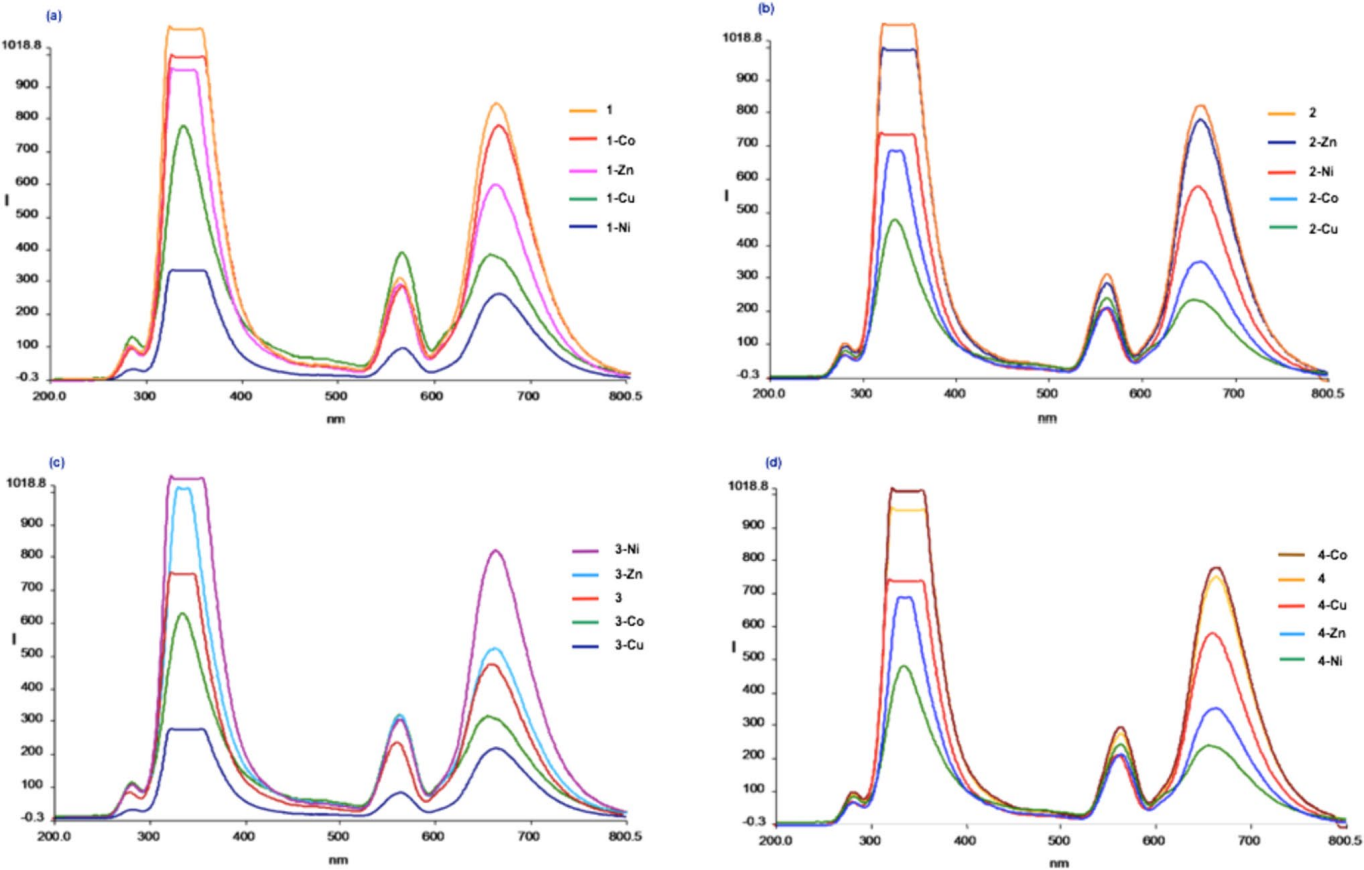
Fluorescence emission spectra of **1**, **2**, **3**, **4** and their metal complexes in DMSO:Acetonitrile (1:1) medium (10^–5^ M, λ_ext_ = 265 nm)

Emission properties of the compounds were also recorded in DMSO:Acetonitrile (ratio 1:1) with the concentration of 1 × 10^–5^ M (Fig. 4). When excitated at 265 nm, all the compounds exhibited triple emission bands (I_max_) at; 339 nm, 564 nm, 675 nm for **1**, **1-Co**, **1-Ni**, **1-Cu**, **1-Zn**; 341 nm, 565 nm, 676 nm for **2**, **2-Co**, **2-Ni**, **2-Cu**, **2-Zn**; 345 nm, 564 nm, 673 nm for **3**, **3-Co**, **3-Ni**, **3-Cu**, **3-Zn**; 342 nm, 564 nm, 675 nm for **4**, **4-Co**, **4-Ni**, **4-Cu**, **4-Zn**. At their excitation wavelength of 265 nm, all molecules exhibited the highest fluorescence emission bands at 675 nm (for **1** and compl.), 676 nm (for **2** and compl.), 673 nm (for **3** and compl.), 675 nm (for **4** and compl.) in near infra-red region. Near-infrared (NIR) fluorescence is a light wavelength of 650–950 nm, and is generally preferred for in vivo fluorescence imaging because of its good tissue penetration and low autofluorescence from adjacent tissues [35, 36].

According to the spectra, the highest emission intensities were observed for the azomethine ligands **1**, **2** and **3** while the other metal complexes had quenchings. **1-Ni**, **2-Cu** and **3-Cu** complexes exhibited the lowest intensities in their own emission spectra. Emission spectra of trimethoxy-ended azomethine ligand **4** and its metal complexes were also investigated and it was observed that **4-Co** exhibited the highest emission compared to **4** and its other metal complexes. **4-Ni** also showed the lowest emission.

The existences of -OCH_3_ methoxy / -OH hydroxy groups with oxygene donor, -CH = N- azomethine group with nitrogen donor and delocalised π-conjugated system in aromatic organic ligands enhances the fluorescence intensities in molecular structures. Quenching of emission may be caused by intersystem crossing, electron exchange, and photoinduced electron transfer. The present quenchings may be due to the complex formation between divalent metal ion and the azomethine fluorophore caused by static PET process [37, 38].

### Antibacterial Activity

**Fig. 5:**
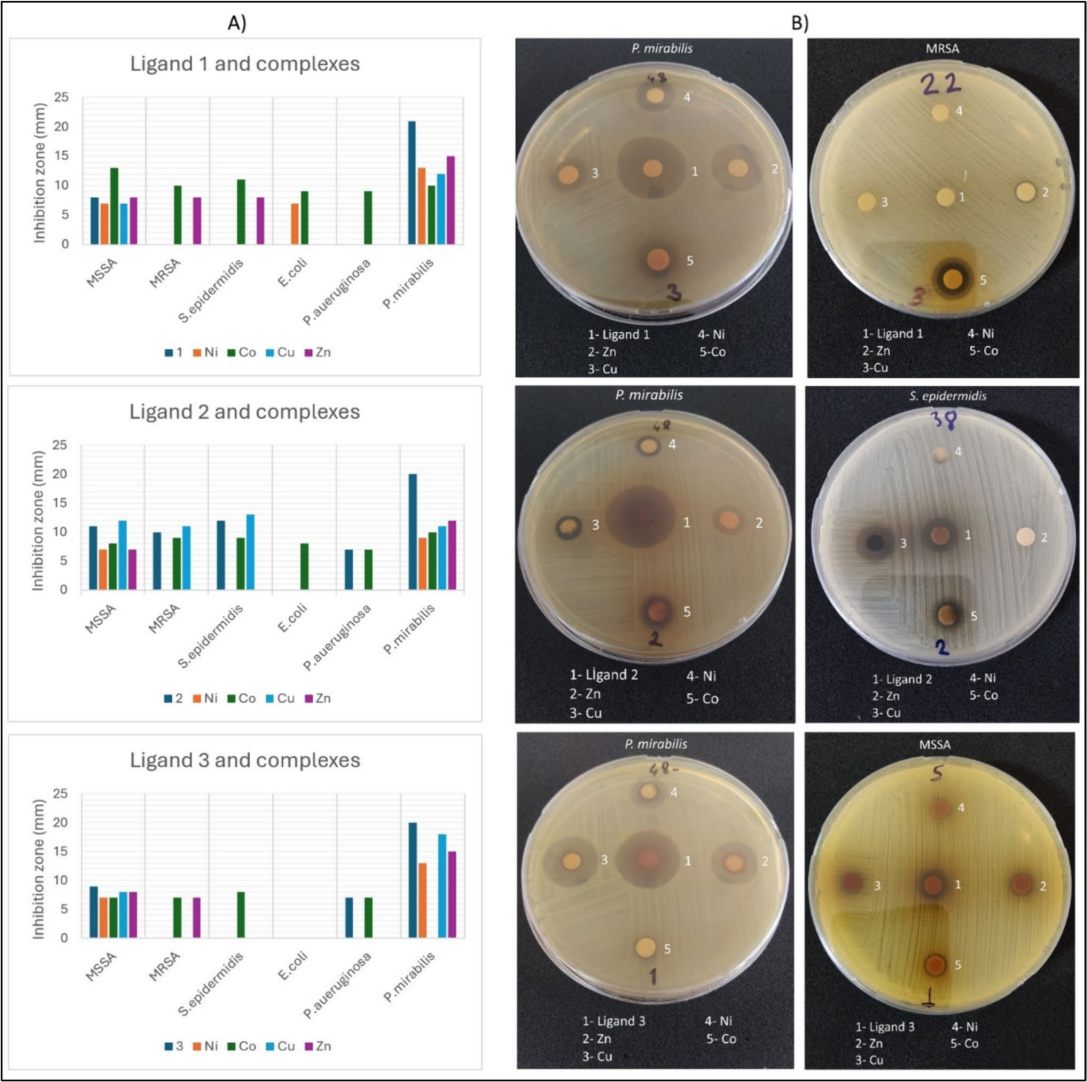
Disc diffusion assay of **1**, **2**, **3** and their complexes. **A** Graphics of Inhibition zones **B)** Some petri images

The antibacterial activity of the synthesized Schiff bases and their metal complexes was evaluated using the disk diffusion method, the broth microdilution method, and antibiofilm screening. The disc diffusion and MIC results are shown in Tables 1 and 2 and Fig. 5. Examining the data, **1**, **2**, and **3** azomethine ligands showed remarkable selective activity (inhibition zone ≥ 20) against the Gram-negative bacteria *Proteus mirabilis*. No bacterial activity was observed for** 4** and its complexes. **1** showed significant efficacy solely against *P. mirabilis*, with a zone diameter of 21 mm. Complexes of the **1** are generally sensitive to *P. mirabilis*, with zone diameters ranging from 10–15 mm. Only the 1-Co complex with a zone diameter of 9–13 mm is effective against tested bacteria other than *P. mirabilis*. **2** was the most efficient ligand against the Gram-positive bacteria, MRSA, and *S. epidermidis*, with zones of 11, 10, and 12 mm, respectively. **2** showed considerable activity specifically against *P. mirabilis* among Gram-negative bacteria, with a zone diameter of 20 mm. **2-Cu** was the most effective of the ligand **2** complexes with zone diameter values of 11–13 mm. **3** exhibited significant antibacterial activity against *P. mirabilis*, with a zone diameter of 20 mm. The **3-Cu** complex is sensitive to *P. mirabilis* with a zone diameter of 18 mm among the ligand-**3** complexes. **3-Ni** and **3-Zn** complexes are moderately sensitive with a 13–15 mm value. When the results of broth microdilution studies performed by selecting ligands and complexes that are sensitive to bacteria were examined, the **2-Cu** complex exhibited the highest efficacy against *S. epidermidis* with a MIC value of 128 µg/mL. The ligands showed the highest sensitivity *to P. mirabilis*, with a MIC value of 256 µg/mL. The MIC values of compounds typically range between 256 and 512 µg/mL. These results indicate that the compounds are low effective compared to the MIC values of the standard antibiotics Cefuroxime (CFR) and Cefazolin (CFZ).

The antibacterial activity of Schiff bases and their metal complexes have been extensively studied and documented in several reviews [18, 39, 40]. Our findings are in agreement with several significant studies in this area. Our study found that azomethines 1, 2, and 3 exhibited remarkable selective activity against the opportunistic gram-negative bacteria *P. mirabilis*. This observation is consistent with the findings of Fatma et al. [41] who reported that Schiff base-metal complexes show enhanced antibacterial activity against *P. mirabilis* due to increased cell membrane permeability facilitated by metal ions. The azomethine group in Schiff bases is responsible for biological properties due to its hydrogen bond formation with cell constituents, limiting normal cell development [42]. Its ability to form complexes with metal through chelation has extraordinary pharmacophore properties, exhibiting better biological activities. Further, chelation increases the lipophilic character of central metal ions, and subsequently, it favors the permeation over the lipid layer of the cell membrane [43]. The increased potency of the 2-Cu complex (zone diameters of 11–13 mm) against various bacteria is particularly noteworthy and is in agreement with findings from Hangan et al. [44], where copper complexes of Schiff bases showed more antibacterial efficacy compared to their ligand counterparts. The varying degrees of antibacterial activity observed among different metal complexes suggest that the nature of the metal ion plays a crucial role in the overall efficacy of the Schiff base complexes. This is corroborated by the study of Sadeek et al. [45], which emphasized that different metal ions can influence the stability, solubility, and overall bioactivity of the complexes. It was also intriguing to us that Azomethine **4** and its complexes exhibited no antibacterial activity. However, the nature of the ligand is quite distinct from other azomethines, particularly due to the absence of an -OH group in the para- position and the presence of -OCH_3_ groups instead. These structural differences can be attributed to limiting the formation of hydrogen bonds, thus reducing the affinity for biological targets [46]. Overall, these findings suggest that the synthesized compounds have the potential as antibacterial agents for combating infections caused by *P. mirabilis*. Further studies are needed to determine the precise mechanism of action and potential for clinical use.

### Antibiofilm Activity

**Fig. 6:**
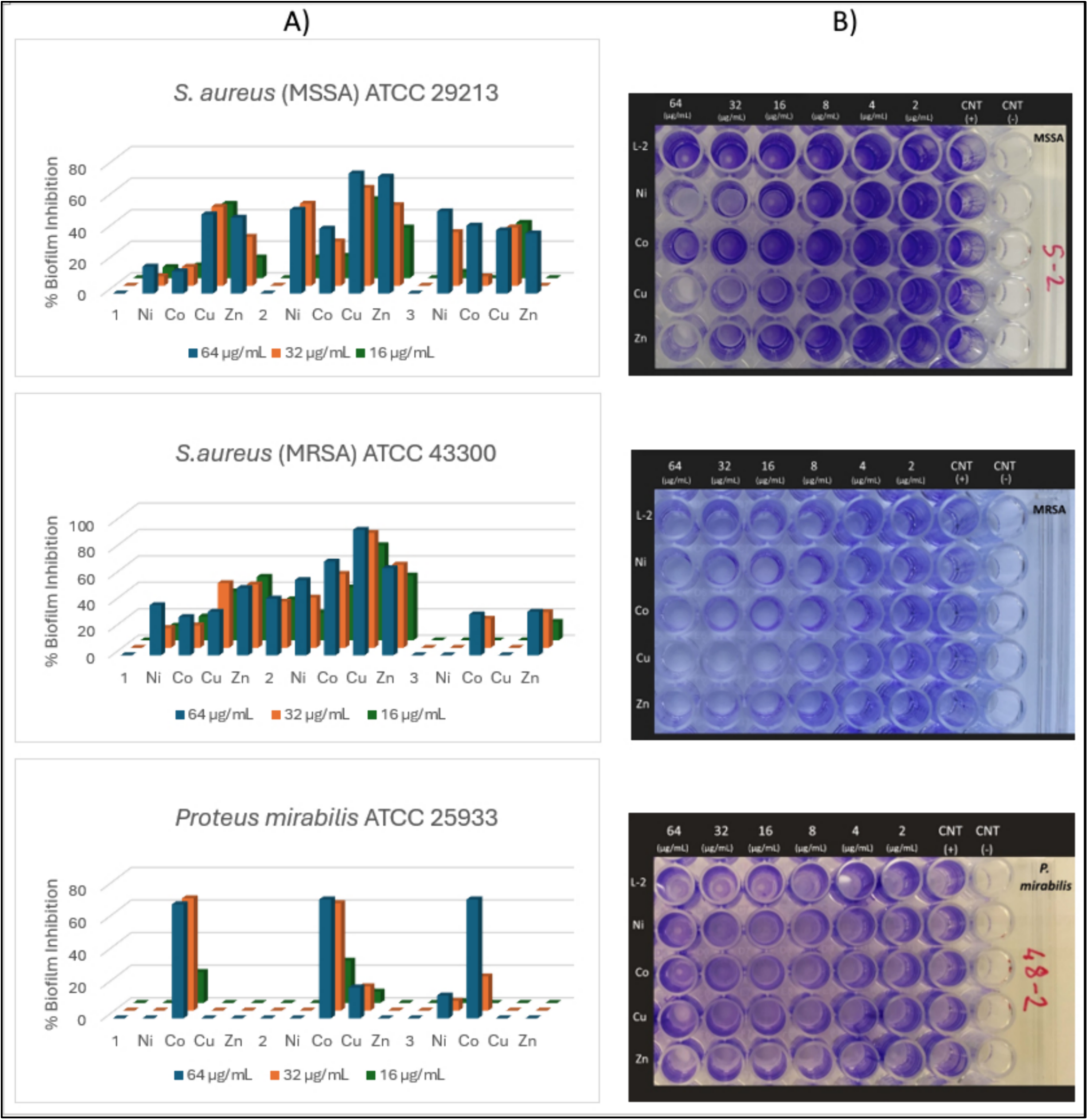
Antibiofilm activity of **1**–**3** and their complexes by Crystal Violet Assay **A)** % Inhibition graphics **B)** Crystal violet staining images of **2** and its complexes

The effects of **1**–**3** and their metal complexes on biofilm formation by MSSA ATCC 29213, MRSA ATCC 43300 and *P. mirabilis* ATCC 25933 were analyzed in terms of total biomass with crystal violet microtitration assay. For this test, bacterial cells were incubated with the compounds at sub-MIC doses during the whole period of biofilm formation (24 h) on a polystyrene microtitration plate. When the % inhibition values (Table 3 and Fig. 6) of compounds were examined, it was observed that ligands did not have a noticeable effect on biofilm formation for any of the strains tested. Metal complexes, on the other hand, had a significant inhibitory effect on biofilm formation, with several of them suppressing biofilms by more than 50% at lower concentrations compared to the ligands. In particular, the metal complexes of **2** exhibited the highest inhibition percentage, with a significant reduction in biofilm formation for MRSA and MSSA. Interestingly, **2-Cu** showed the highest inhibition, surpassing 72% even at low concentrations (16 μg/mL) for MRSA. The inhibition percentage continued to increase as the concentration of **2-Cu** increased and reached 95% at 64 µg/ml. In the same way, **2-Cu** demonstrated valuable antibiofilm activity for *Staphylococcus spp.* by decreasing the production of MSSA biofilms by more than 50%. In addition, other complexes of **2** have received attention as potential antibiofilm agents, particularly in preventing the formation of MRSA and MSSA biofilms, by showing inhibitions of over 50%. However, the biofilms formed by the Gram-negative bacteria *P. mirabilis* were only influenced by the Co complexes of azomethines, with inhibitory percentages above 70% at 64 mg/ml. This specificity suggests that the Co complexes may target a specific component or pathway in the biofilm formation process of *P. mirabilis*. Further research is needed to understand the mechanisms underlying selective inhibition and to investigate the potential of these Co complexes as novel antibiofilm agents.

**Table 3:** Biofilm inhibiting values (%)

	*S.aureus* (MSSA) ATCC 29213			*S.aureus* (MRSA) ATCC 43300			*P. mirabilis* ATCC 25933		
	64 µg/mL	32 µg/mL	16 µg/mL	64 µg/mL	32 µg/mL	16 µg/mL	64 µg/mL	32 µg/mL	16 µg/mL
1	-	-	-	-	-	-	-	-	-
Ni	17	6	7	38	15	11	-	-	-
Co	14	12	8	29	17	18	70	69	19
Cu	50	50	47	33	49	37	-	-	-
Zn	48	31	13	51	48	48	-	-	-
2	-	-	-	43	35	31	-	-	-
Ni	53	52	13	57	38	21	-	-	-
Co	41	28	14	71	56	40	73	66	26
Cu	76	62	50	95	87	72	19	15	7
Zn	74	51	32	66	63	49	-	-	-
3	-	-	-	-	-	-	-	-	-
Ni	52	34	4	-	-	-	14	6	-
Co	43	6	-	31	22	-	73	21	-
Cu	40	37	35	-	-	-	-	-	-
Zn	38	-	-	33	27	14	-	-	-

In recent years, there has been considerable interest in the use of complexes to combat biofilm-associated infections [44]. These complexes can target key components of biofilms, such as extracellular polymeric substances (EPS), disrupting their formation. Additionally, complexes have been found to disrupt the quorum-sensing mechanisms that allow bacteria to communicate and coordinate within biofilms. By interfering with these communication pathways, complexes can hinder the formation and stability of biofilms, making them more susceptible to traditional antibiotics. Several reviews of metal complexes have highlighted their potential to target and disrupt biofilms formed by Gram-positive and Gram-negative bacteria [19, 47, 48]. The biological activity of the complexes is substantially higher than that of ligands or metals alone, emphasizing the importance of complex formation [49, 50]. Notably, copper (II) complexes may possess chemical nuclease activity [51], and various researchers have synthesized and evaluated complexes that play a role in DNA transformation [52, 53]. For instance, one study found that copper (II) complexes demonstrated effectiveness against both Gram-positive and Gram-negative bacteria, with a tendency for higher activity against the former. Strikingly, the copper complexes evaluated showed significant antibiofilm activity against a clinical isolate of methicillin-resistant Staphylococcus aureus (MRSA) and were shown to be more effective at reducing biofilms than vancomycin, an antibiotic commonly used to treat MRSA infections [50].

Overall, the findings of our study indicate that metal complexes have stronger antibiofilm activities compared to azomethines. When targeting MSSA and multidrug-resistant MRSA biofilms, the copper (II) complex of **2** stands out for its antibiofilm properties. Moreover, the selective inhibition of the cobalt (II) complexes of azomethines on the opportunistic pathogen *Proteus mirabilis* biofilms suggests that metal complexes might be promising candidates for antibiofilm research.

## Conclusion

Herein, π-conjugated azomethine molecules with hydroxy-substitutions and their divalent metal complexes were reported. Azomethines were generated from the condensation of primary amines and active carbonyl group of aldehyde species by nucleophilic addition with the result of a dehydration. Metal complexations were performed in methanol medium with divalent metal acetate salts. Their promising fluorescence behaviours were observed in photophysical investigations. Antibacterial and antibiofilm properties of π-conjugated azomethines against clinically important bacteria such as MSSA, MRSA and *P. mirabilis* were investigated. Results show that ligands and complexes have a significant antibacterial effect, particularly on *Proteus mirabilis*. Antibiofilm study reveals that metal complexes have stronger antibiofilm activities than ligands, with the copper (II) complex of **2** being particularly effective against MSSA and MRSA biofilms. Additionally, selective inhibition of cobalt (II) complexes on *Proteus mirabilis* biofilms suggests that metal complexes have the potential to be developed as effective antibiofilm agents against a range of bacteria. Further research into the mechanisms of action of these metal complexes on different types of biofilms could provide valuable insights for the development of novel antibiofilm strategies.

## Supplementary Information

Below is the link to the electronic supplementary material.

Supplementary file1 (DOCX 9343 KB)

## Data Availability

No datasets were generated or analysed during the current study.
