# Supplementary material for: Highly Fluorescent π-Conjugated Azomethines and Divalent Metal Complexes as Antibacterial and Antibiofilm Nominees
Source: J Fluoresc. 2024 Jul 30;35(6):4673–88. doi: 10.1007/s10895-024-03855-x (PMC12206175; doi:10.1007/s10895-024-03855-x)
Supplement: Supplementary file 1 — Supplementary file1 (DOCX 9343 KB) [file 10895_2024_3855_MOESM1_ESM.docx]

**HIGHLY FLUORESCENT π-CONJUGATED AZOMETHINES AND DIVALENT METAL COMPLEXES AS ANTIBACTERIAL AND ANTIBIOFILM NOMINEES**

^1^Şeyma Nur URAL BAYDENİZ, ^1^Halil İsmet UÇAN, ^2^Fatih SEVGİ, ^3^İhsan OBALI, ^1^Aslıhan YILMAZ OBALI

^1^Department of Chemistry, Science Faculty, Selcuk University, Turkey

^2^Department of Medical Services and Techniques, Vocational School of Health Services, Selcuk University, Turkey

^3^Department of Biology, Science Faculty, Selcuk University, Turkey

**SUPPLEMENTARY FILE; FT-IR, ^1^H-NMR AND ^13^C-NMR SPECTRA OF COMPOUNDS**

**Fig.S1.** FT-IR Spectra of free ligand **1**.

**Fig.S2.** FT-IR Spectra of **1-Co**.

**Fig.S3.** FT-IR Spectra of **1-Ni**.

**Fig.S4.** FT-IR Spectra of **1-Cu**.

**Fig.S5.** FT-IR Spectra of **1-Zn**.

**Fig.S6.** FT-IR Spectra of free ligand **2**.

**Fig.S7.** FT-IR Spectra of **2-Co**.

**Fig.S8.** FT-IR Spectra of **2-Ni**.

**Fig.S9.** FT-IR Spectra of **2-Cu**.

**Fig.S10.** FT-IR Spectra of **2-Zn**.

**Fig.S11.** FT-IR Spectra of free ligand **3**.

**Fig.S12.** FT-IR Spectra of **3-Co**.

**Fig.S13.** FT-IR Spectra of **3-Ni**.

**Fig.S14.** FT-IR Spectra of **3-Cu.**

**Fig.S15.** FT-IR Spectra of **3-Zn**.

**Fig.S16.** FT-IR Spectra of free ligand **4**.

**Fig.S17.** FT-IR Spectra of **4-Co**.

**Fig.S18.** FT-IR Spectra of **4-Ni**.

**Fig.S19.** FT-IR Spectra of **4-Cu**.

**Fig.S20.** FT-IR Spectra of **4-Zn**.

**
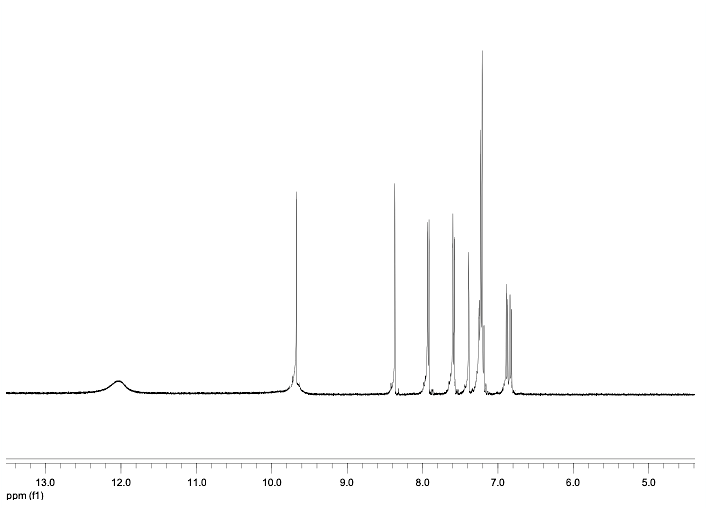
**

**Fig.S21.** ^1^H-NMR Spectra of free ligand **1**.


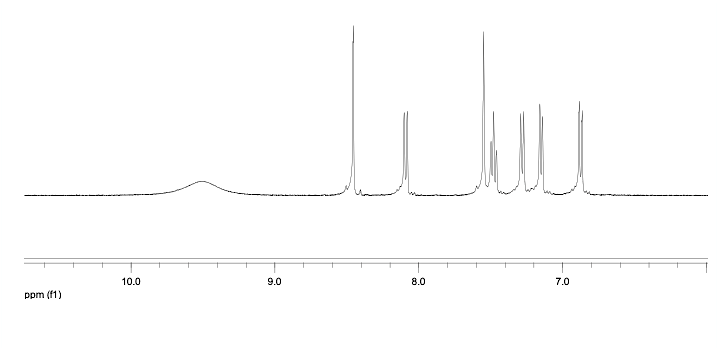


**Fig.S22.** ^1^H-NMR Spectra of free ligand **2**.


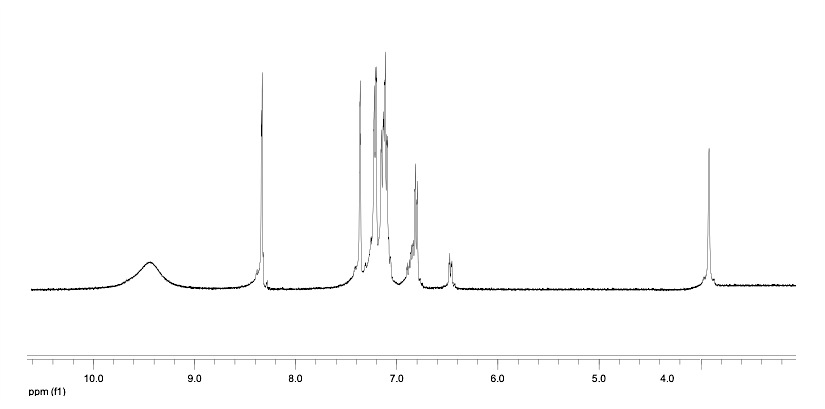


**Fig.S23.** ^1^H-NMR Spectra of free ligand **3**.


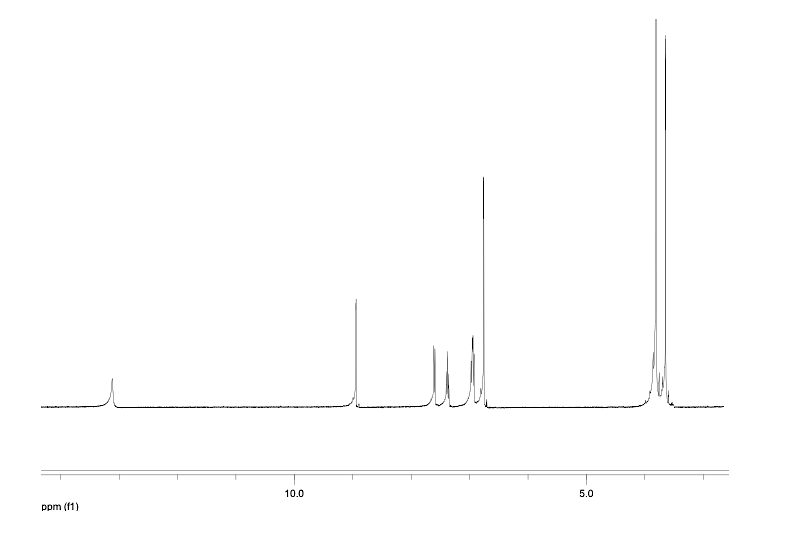


**Fig.S24.** ^1^H-NMR Spectra of free ligand **4**.


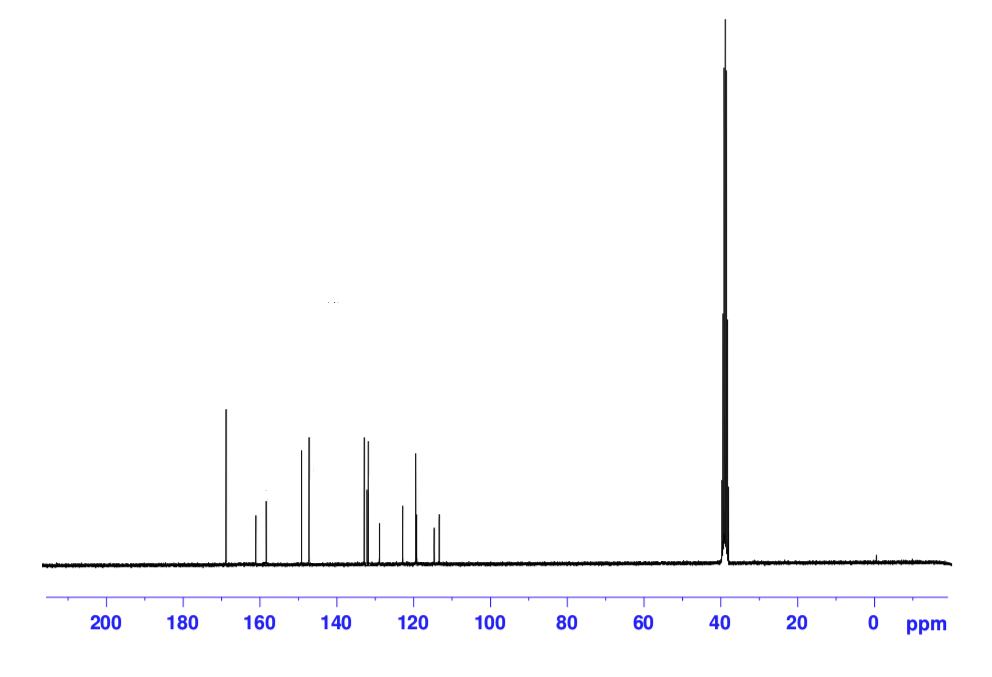


**Fig.S25.** ^13^C-NMR Spectra of free ligand **1**.


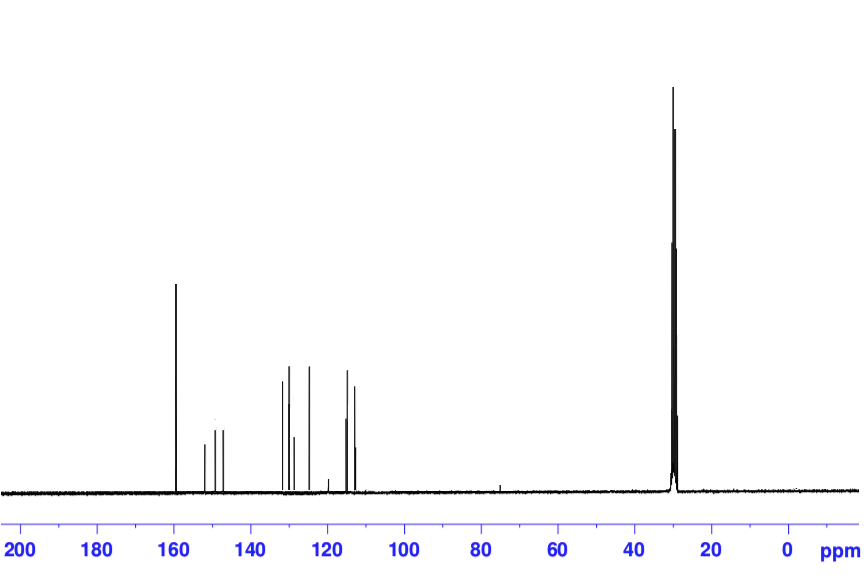


**Fig.S26.** ^13^C-NMR Spectra of free ligand **2**.


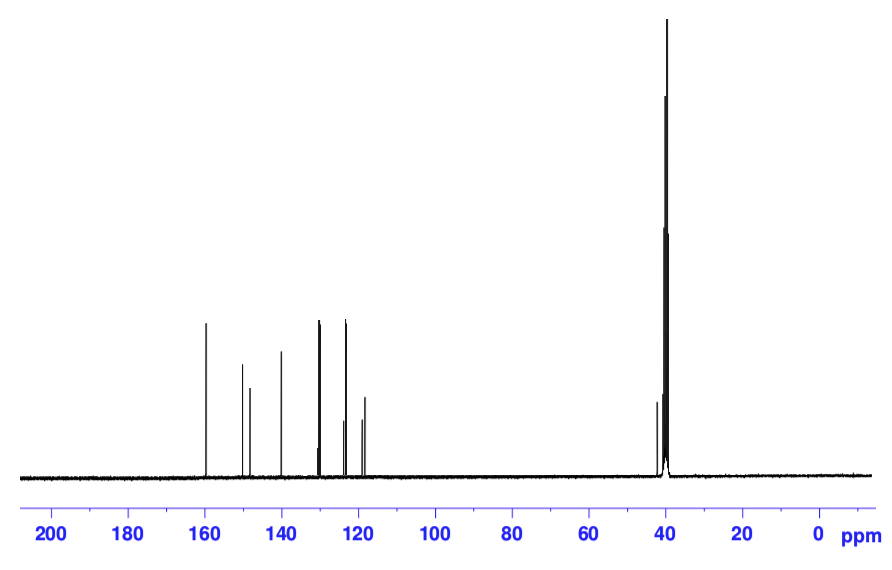


**Fig.S27.** ^13^C-NMR Spectra of free ligand **3**.


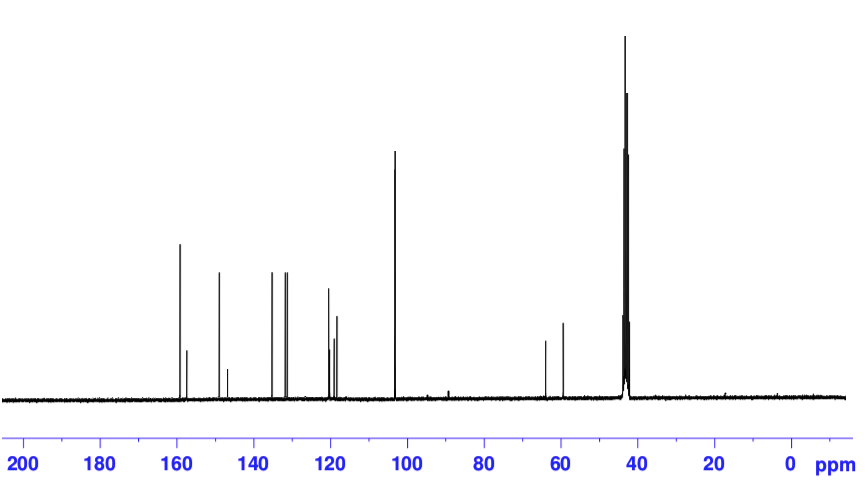


**Fig.S28.** ^13^C-NMR Spectra of free ligand **4**.
